# Supplementary material for: Development of a General Health Score Based on 12 Objective Metabolic and Lifestyle Items: The Lifestyle and Well-Being Index
Source: Healthcare (Basel). 2022 Jun 11;10(6):1088. doi: 10.3390/healthcare10061088 (PMC9222586; doi:10.3390/healthcare10061088)
Supplement: Supplementary file 1 [file healthcare-10-01088-s001.zip › Supporting Files 1.pdf]

Supplementary Table S1 – Individual R2 estimates from linear regression models and p-values for logistic models for each the items included in the LWB-I.

Selected Items	Outcome			
	Modified SF-36 score		Poor + Fair SPH	Excellent SPH
	Individual R-squared	P-Value	P-Value	P-Value
Have you felt downhearted and blue? (Item 28, SF-36)	0.3128	< 0.001	< 0.001	< 0.001
Did you feel tired? (Item 31, SF-36)	0.1304	< 0.001	< 0.001	< 0.001
Insomnia	0.0123	< 0.001	< 0.001	< 0.001
Age (years)	0.0043	< 0.001	< 0.001	< 0.001
Number of Pre-existing Diseases[a]	0.0022	< 0.001	< 0.001	< 0.001
Physical activity[a]	0.001	< 0.001	0.0221	< 0.001
BMI categories[a]	0.0006	< 0.001	0.0052	< 0.001
Sex (female vs. male)	0.0012	< 0.001	0.7147	0.0101
Smoking status	0.0004	0.0009	0.0624	0.0517
Fruits + vegetables (for each serv./d)	0.0002	0.0203	0.2381	0.1304
Family History of Disease[a]	0.0002	0.0251	0.0039	0.0109
Sugary products[a] (serv./d)	0.0001	0.0566	0.0401	0.454

Items are arranged in decreasing order according to their individual contribution to the R^2 of the model. Each item was included as described in the main text into a nested model in the order in which they are presented in this table. Linear regression models using the modified version of the SF-36 as an outcome were used to obtain individual R^2 values. For binary outcomes, nested logistic regression models were performed using the dichotomic variables for the pooled categories of "poor" and "fair" and in a separate model, those with "excellent" self-perceived health. Likelihood ratio tests were performed and reported as p-values, which represent the significance of the inclusion of each item into the nested model. a: Refer to main text for variable description.

Supplementary Table S2 - Sample characteristics according to the Lifestyle and Well-being index. Cut-off points using 75 pts as the lower cut-off and 81 pts as the upper cut-off.

Characteristics	LWB Index (80-86 Cut-off points)			P-Value
	**Poor (<80 pts) (n=4,583)**	**Transition (80-86 pts) (n=4,661)**	**Excellent (>86 pts) (n=5,924)**	
Sex (%) Female	68.3	61.3	52.0	< 0.001
Age (years)	38.4 (12.0)	38.8 (12.1)	38.1 (12.0)	< 0.007
BMI (kg/m^2)	23.6 (3.8)	23.6 (3.6)	23.5 (3.2)	0.062
Smoking status (%)				< 0.001
Never	46.0	45.6	54.8	
Current	23.9	22.2	18.4	
Former	30.1	32.2	26.8	
Family history of diseases[a] (%)				< 0.001
0	32.5	36.2	38.6	
1	44.5	42.5	44.1	
2	23.1	21.3	17.4	
Pre-existing diseases[b] (%)				< 0.001
0	71.4	73.0	79.4	
1	21.6	20.7	17.8	
2	6.1	5.8	2.5	
3	0.9	0.5	0.3	
Insomnia (%)				< 0.001
Never	20.6	27.4	49.8	
Rarely	47.2	52.4	43.7	
Yes	32.3	20.2	6.5	
Physical Activity (METs-h/week)	18.5 (20.4)	20.7 (21.0)	25 (24.9)	< 0.001
Fruits + vegetables (serv/day)	4.6 (2.8)	4.6 (2.6)	4.8 (3.0)	< 0.001
Added sugars[c] (%)				0.001
None	5.2	5.1	5.8	
< 1 serv/day	91.4	91.8	92.2	
> 1 serv/day	3.4	3.1	2.1	
Have you felt downhearted and blue? (Item 28, SF-36)				<0.001
Most to All of the time	13.9	0.0	0.0	
Some of the time	49.1	3.5	0.1	
A little of the time	30.5	76.4	36.3	
None of the time	6.6	20.1	63.6	
Did you feel tired? (Item 31, SF-36)				<0.001
Most to All of the time	55.0	3.7	0.1	
Some of the time	37.5	70.7	13.2	
A little of the time	7.2	24.7	72.6	
None of the time	0.3	0.9	14.1	

Subjects with a LWB-I score below 75 points were categorized as having *poor* LWB, those between 75 and 81 are considered in *transition*, and individuals above 81 points are considered to have excellent

LWB. Data is presented as means (SD), unless otherwise stated Units of measurement are presented along with each variable Categorization of the LWB index was done according to the cut-offs obtained through Youden's index P values were obtained using χ^2 distribution for categorical variables and one-way ANOVAs for continuous variables. Prior assessment of data distribution of continuous variables was analyzed using tests for normality and graphical means. Abbreviations BMI: Body mass index; SF-36: Short Form 36 Questionnaire, SF-33: Modified version of the SF-36 excluding items 1, 10 and 28. a: identifies the existence of chronic diseases in both parents ranging from absent (0), present in one parent (1), and present in both parents (2). A detailed list of the included diseases can be found in the main text. b: Identifies the number of diseases present for each subject including: diabetes, hypertension, and hypercholesterolemia. c: Pooled consumption of standard servings of carbonated beverages (200cc), sugar (10g) and marmalade (10g) were included.

Supplementary Table S3 – Subgroup analysis for individuals with BMI ≥ 30 kg/m^2. β-Estimations from the linear model.

**Characteristics**	**Beta Coefficient (β)**	**CI: 95%**		**P-Value**
		**Lower Bound**	**Upper Bound**	
Sex (female vs. male)	-3.734	-5.511	-1.958	< 0.001
Age (for each year)	-.013	-.085	.058	.72
BMI Category				
Underweight (<18.5 kg/m^2)	-	-	-	-
Normal (18.5-24.9 kg/m^2)	-	-	-	-
Overweight (25.0-29.9 kg/m^2)	-	-	-	-
Obesity (>30.0 kg/m^2)	-	-	-	-
Smoking status				
Never	0 (Ref.)	0 (Ref.)	0 (Ref.)	Ref.
Current	-.438	-2.481	1.606	.674
Former	-.051	-1.805	1.704	.955
Family History of Disease[a]				
0	0 (Ref.)	0 (Ref.)	0 (Ref.)	Ref.
1	.692	-1.265	2.649	.488
2	.507	-1.54	2.554	.627
Number of Pre-existing Diseases				
0	0 (Ref.)	0 (Ref.)	0 (Ref.)	Ref.
1	-2.229	-3.943	-.514	.011
2	-4.759	-7.038	-2.48	< 0.001
3	-2.652	-7.482	2.178	.281
Insomnia				
Never	0 (Ref.)	0 (Ref.)	0 (Ref.)	Ref.
Rarely	-.958	-2.677	.761	.274
Yes, currently or in the past	-3.908	-6.068	-1.749	0
Physical Activity				
Below recommendations[#]	-.793	-2.293	.707	.3
Recommended[#]	0 (Ref.)	0 (Ref.)	0 (Ref.)	Ref.
Above recommendations[#]	-.008	-3.55	3.533	.996

Fruits + vegetables (for each serv./d)	.037	-.198	.271	.758
Sugary products[b] (serv./d)				
None	0 (Ref.)	0 (Ref.)	0 (Ref.)	Ref.
Less than 1	.765	-1.91	3.441	.574
More than 1	-4.225	-9.067	.617	.087
Have you felt downhearted and blue? (Item 28, SF-36)				
All of the time	-25.321	-35.964	-14.678	< 0.001
Most of the time	-12.941	-20.416	-5.466	.001
A good bit of time	-21.916	-26.218	-17.614	< 0.001
Some of the time	-13.021	-15.427	-10.615	< 0.001
A little of the time	-4.336	-6.026	-2.646	< 0.001
None of the time	0 (Ref.)	0 (Ref.)	0 (Ref.)	Ref.
Did you feel tired? (Item 31, SF-36)				
All of the time	-36.441	-44.829	-28.052	< 0.001
Most of the time	-30.01	-35.269	-24.75	< 0.001
A good bit of time	-15.095	-18.799	-11.392	< 0.001
Some of the time	-7.674	-10.688	-4.661	< 0.001
A little of the time	-3.109	-6.097	-.122	.041
None of the time	0 (Ref.)	0 (Ref.)	0 (Ref.)	Ref.

The coefficients used to develop the index were obtained through multivariate linear regressions using the "*Leave one out*" method. The modified version of the SF-36 (SF-33) was used as a dependent (predicted) variable, whereas the variables described in this table were included as independent variables in a single model. For categorical variables, reference categories were set to the absence of the condition or defined by literature-based recommendations. Each β-Coefficient represents the pondered association between each variable or variable category and an individual's lifestyle and well-being a: The item designates the number of parents that present any of the diseases described in the main text. b: Pooled analysis of standard servings of sodas, including artificially sweetened beverages (200cc), sugar (10g) and marmalade (10g) were included. #:Below recommendations - less than 2.5 h/wk of moderate intensity activities; Recommended - 2.5 and 5 h/wk of moderate/vigorous intensity activities; Above recommendations - over 5 h/wk of vigorous physical activity.

Supplementary Table S4 - Subgroup analysis for individuals with age ≥ 50 years. β-Estimations from the linear model.

Characteristics	Beta Coefficient (β)	CI: 95%		P-Value
		Lower Bound	Upper Bound	
Sex (female vs. male)	-.797	-1.607	.013	.054
Age (for each year)	-	-	-	-
BMI Category				
Underweight (<18.5 kg/m^2)	1.929	-2.217	6.075	.362
Normal (18.5-24.9 kg/m^2)	0 (Ref.)	0 (Ref.)	0 (Ref.)	Ref.
Overweight (25.0-29.9 kg/m^2)	-.848	-1.613	-.084	.03
Obesity (>30.0 kg/m^2)	-1.478	-2.741	-.216	.022

Smoking status				
Never	0 (Ref.)	0 (Ref.)	0 (Ref.)	Ref.
Current	.085	-.948	1.118	.871
Former	-.665	-1.437	.108	.092
Family History of Disease[a]				
0	0 (Ref.)	0 (Ref.)	0 (Ref.)	Ref.
1	-0.18	-0.89	0.52	0.611
2	-0.18	-0.97	0.61	0.649
Number of Pre-existing Diseases				
0	0 (Ref.)	0 (Ref.)	0 (Ref.)	Ref.
1	-0.29	-0.93	0.36	0.383
2	-2.40	-3.28	-1.53	< 0.001
3	-4.61	-6.73	-2.48	< 0.001
Insomnia				
Never	0 (Ref.)	0 (Ref.)	0 (Ref.)	Ref.
Rarely	-1.149	-1.992	-.306	.008
Yes, currently or in the past	-3.182	-4.173	-2.19	< 0.001
Physical Activity				
Below recommendations[#]	-.648	-1.4	.103	.091
Recommended[#]	0 (Ref.)	0 (Ref.)	0 (Ref.)	Ref.
Above recommendations[#]	-.703	-1.944	.538	.267
Fruits + vegetables (for each serv./d)	-.065	-.177	.047	.256
Sugary products[b] (serv./d)				
None	0 (Ref.)	0 (Ref.)	0 (Ref.)	Ref.
Less than 1	-.105	-1.164	.954	.846
More than 1	-1.528	-3.646	.59	.157
Have you felt downhearted and blue? (Item 28, SF-36)				
All of the time	-17.387	-22.869	-11.906	< 0.001
Most of the time	-22.03	-26.362	-17.698	< 0.001
A good bit of time	-22.621	-24.916	-20.326	< 0.001
Some of the time	-10.898	-12	-9.796	< 0.001
A little of the time	-3.61	-4.392	-2.827	< 0.001
None of the time	0 (Ref.)	0 (Ref.)	0 (Ref.)	Ref.
Did you feel tired? (Item 31, SF-36)				
All of the time	-40.553	-45.713	-35.393	< 0.001
Most of the time	-28.549	-31.416	-25.682	< 0.001
A good bit of time	-17.059	-18.602	-15.516	< 0.001
Some of the time	-8.95	-10.15	-7.75	< 0.001
A little of the time	-4.042	-5.179	-2.905	< 0.001
None of the time	0 (Ref.)	0 (Ref.)	0 (Ref.)	Ref.

The coefficients used to develop the index were obtained through multivariate linear regressions using the "*Leave one out*" method. The modified version of the SF-36 (SF-33) was used as a dependent (predicted) variable, whereas the variables described in this table were included as independent variables in a single model. For categorical variables, reference categories were set to the absence of the condition or defined by literature-based recommendations. Each β-Coefficient represents the pondered association between each variable or variable category and an individual's lifestyle and well-being a: The item designates the number of parents that present any of the diseases described in the main text. b: Pooled analysis of standard servings of sodas, including artificially sweetened beverages (200cc), sugar (10g) and marmalade (10g) were included. #:Below recommendations - less than 2.5 h/wk of moderate intensity activities; Recommended - 2.5 and 5 h/wk of moderate/vigorous intensity activities; Above recommendations - over 5 h/wk of vigorous physical activity.
